# The metabolism of primaquine to its active metabolite is dependent on CYP 2D6

**DOI:** 10.1186/1475-2875-12-212

**Published:** 2013-06-20

**Authors:** Brandon S Pybus, Sean R Marcsisin, Xiannu Jin, Gregory Deye, Jason C Sousa, Qigui Li, Diana Caridha, Qiang Zeng, Gregory A Reichard, Christian Ockenhouse, Jason Bennett, Larry A Walker, Colin Ohrt, Victor Melendez

**Affiliations:** 1 Division of Experimental Therapeutics, Walter Reed Army Institute of Research, 503 Robert Grant Ave, Silver Spring, MD 20910, USA; 2 National Center for Natural Products Research and Department of Pharmacology, School of Pharmacy, University of Mississippi, Oxford, MS 38677, USA

## Abstract

**Background:**

The efficacy of the 8-aminoquinoline (8AQ) drug primaquine (PQ) has been historically linked to CYP-mediated metabolism. Although to date no clear evidence exists in the literature that unambiguously assigns the metabolic pathway or specific metabolites necessary for activity, recent literature suggests a role for CYP 2D6 in the generation of redox active metabolites.

**Methods:**

In the present study, the specific CYP 2D6 inhibitor paroxetine was used to assess its effects on the production of specific phenolic metabolites thought to be involved in PQ efficacy. Further, PQ causal prophylactic (developing liver stage) efficacy against *Plasmodium berghei* in CYP 2D knockout mice was assessed in comparison with a normal C57 background and with humanized CYP 2D6 mice to determine the direct effects of CYP 2D6 metabolism on PQ activity.

**Results:**

PQ exhibited no activity at 20 or 40 mg/kg in CYP 2D knockout mice, compared to 5/5 cures in normal mice at 20 mg/kg. The activity against developing liver stages was partially restored in humanized CYP 2D6 mice.

**Conclusions:**

These results unambiguously demonstrate that metabolism of PQ by CYP 2D6 is essential for anti-malarial causal prophylaxis efficacy.

## Background

The 8-aminoquinoline anti-malarial drug primaquine (PQ) is of seminal importance in the fight against malaria, as it is the only drug currently indicated to treat relapsing strains of *Plasmodium vivax* and *Plasmodium ovale* (antihypnozoite activity). Due to its antihypnozoite in *P*. *vivax* and gametocytocidal activity in *P*. *falciparum*, it is often considered in strategies for mass administration with the goal of malaria elimination [1].

PQ efficacy is thought to be dependent upon biotransformation, but the essential pathways for this activation have, to date, not been reported [2,3]. PQ is known to interact with several CYP enzymes as well as monoamine oxidases (MAOs) [4-7]. Constantino *et al*. [4] demonstrated that the role of MAOs was most likely in the catalysis of the first step (deamination to the aldehyde) on the pathway to carboxyprimaquine, the major plasma metabolite of PQ. Carboxyprimaquine has been shown to lack efficacy or toxicity [4]. However, the most likely mechanism of action for PQ is one mediated by the formation of reactive oxygen species through redox cycling of hydroxylated metabolites and subsequent toxicity to the parasite [2,7,8]. It was recently demonstrated that hydroxy metabolites of PQ are predominantly generated via metabolism by CYP 2D6 [6]. CYP 2D6 is subject to highly polymorphic genetic variability, which affects the pharmacokinetics of roughly 50% of the drugs on the market [9]. If PQ efficacy is solely dependent upon CYP 2D6 metabolism, this could present a serious problem for eradication efforts centred around PQ use, as many populations throughout the world (including endemic areas) have high prevalence of allelic frequency for poor and intermediate activity CYP 2D6 [10]. As an example, Bennett *et al*. recently reported two clinical PQ failures in a *P*. *vivax* challenge which were linked to subjects of the poor and intermediate CYP 2D6 genotype (personal communication by Bennett et al.). The recent development of a murine model of CYP 2D6 polymorphism has made it feasible to address the question of whether PQ efficacy has any pharmacogenomic dependence. Scheer *et al*. created mouse strains in which the CYP 2D cluster is deleted and can be replaced with allelic variants of human CYP 2D6 [11]. In the present study, this model was used to determine if manipulation of CYP 2D mediated metabolic pathways of PQ in mice has direct effects on causal prophylactic anti-malarial efficacy. Specifically, PQ efficacy was compared in strains of mice, which would model either the CYP 2D6 null/poor metabolizer variant or the extensive metabolizer. Further, the effects of the potent CYP 2D6 inhibitor paroxetine (PXT) were demonstrated on the *in vitro* production by human recombinant CYP 2D6 of the phenolic metabolites thought responsible for PQ activity.

## Methods

### Chemicals used

Chemicals used were: primaquine (Sigma, St Louis, MO, USA, #160393), paroxetine (Sigma, #P9623), nicotinamide adenine dinucleotide phosphate, oxidized form (NADP) (Sigma, # 077 K7000), acetonitrile (Fisher Scientific, Waltham, MA, USA, #972970), glucose-6-phosphate (G6P) (Sigma, # 046 K3779), glucose-6-phosphate dehydrogenase (G6PD) (Sigma, # 068 K3795), and magnesium chloride (MgCl_2_) (Sigma, #102 K0154). Mobile phases were made with HPLC grade water, acetonitrile and formic acid.

### CYP2D6 incubations

*In vitro* metabolism studies with the CYP2D6 isoenzyme were conducted according to the manufacturer’s instructions (BD Gentest, San Jose, CA, USA). Briefly, the procedure was as follows: a 30 μl aliquot of 5 mg/ml CYP2D6 was mixed with the NADPH regeneration system A (50 μl) and B (10 μl), and 990 ml of phosphate buffer (pH 7.4, 100 mM) was added. The solution was mixed gently by pipetting and incubated at 37°C for 2 min. Primaquine (10 μM final concentration) was added in the absence or presence of various concentrations of the CYP2D6 inhibitor paroxetine (0, 5, 10, 15, 50 μM). A portion of the mixture (120 μl) was then collected at several time points (0, 60 min) followed by quenching with an equal volume of acetonitrile. The samples were vortexed for 30 sec, and centrifuged at 13,200 × rpm at 4°C for 10 min. The supernatant was collected and loaded onto 96-well plates (200 μl/well) for LC-MS analysis.

### Primaquine metabolite identification

Primaquine samples were analysed using a Waters (Milford, MA, USA) Acquity UPLC system coupled to a Xevo Q-ToF mass spectrometer equipped with a standard electrospray ionization source. Chromatographic separations were achieved using a Waters Acquity BEH C18 1.7 μm 2.1 mm × 100 mm column with a 2 to 98% acetonitrile gradient over 6.10 min at a flow rate of 0.70 mL/min. Mobile phase A consisted of 10 mM ammonium bicarbonate and mobile phase B consisted of acetonitrile. The gradient consisted of phase B increasing from 2 to 60% in the time period of 0 to 2.9 min, followed by 60 to 98% from 2.9 to 4.7 min, holding at 98% B from 4.7 to 5.2 min, and then returning to 2% B from 5.2 to 6.1 min. MS conditions were optimized for primaquine detection in the positive electrospray mode with the corresponding instrumental parameters: capillary 1 kV, sampling cone 20 V, extraction cone 4 V, source temperature 120°C, desolvation temperature 150°C, cone gas flow 30 L/Hr, and desolvation gas flow 600 L/Hr. Low energy MS scans were conducted using a collision energy of 6 V. Primaquine fragments were produced using the MS^E^ mode with a collision energy ramp from 15–18 V. Primaquine metabolites were indentified and analysed using Waters Metabolynx software, MS^E^ and MS/MS analysis.

### IVIS study for C57BL/6 and knockout mice

PQ was administered orally on days −1, 0, and 1 with respect to sporozoite inoculation. At 24, 48, and 72 hours post-sporozoite infection, all inoculated mice were tested using the Caliper Life Sciences (Hopkinton, MA, USA) IVIS Spectrum instrument. Additionally, emerging blood stage infections were measured by a flow cytometry system (FC500 MPL, Beckman Coulter, Miami, FL, USA). Positive and negative controls are routinely used for the IVIS calibration in each test.

### Sporozoites, inoculation and viability check

*Plasmodium berghei* sporozoites (luciferase expressing) were obtained from laboratory-reared female *Anopheles stephensi* mosquitoes from Department of Mosquito Biology, WRAIR and maintained at 18°C for 17 to 22 days after feeding on malaria infected Swiss CD-1/ICR mice. Salivary glands were extracted from malaria-infected mosquitoes and sporozoites were recovered by using in house procedures. Briefly, mosquitoes were separated into abdomen and head/thorax. Heads and thoraxes were triturated with a mortar and pestle and suspended in medium RPMI 1640 containing 1% C57BL/6 mouse serum (Rockland Co., Gilbertsville, PA, USA). A total of 50–80 heads with glands were placed into a 0.5 ml Osaki tube on top of glass wool with enough dissection media to cover the heads. The Osaki tube was kept on ice until all mosquitoes had been dissected. Sporozoites isolated from the same batch of mosquitoes were inoculated into C57BL/6, 2DKO and 2DKO/KI C57BL/6 mice on the same day to control for biological variability in sporozoite preparations. Each mouse was inoculated intravenously in the tail vein with approximately 10,000 sporozoites suspended in 0.1 ml volume on day 0.

To ensure that inoculated sporozoites were viable following the isolation procedure, they were stained with a vital dye containing fluorescein diacetate (50 mg/ml in acetone) and ethidium bromide (20 μg/ml in phosphate-buffered saline; Sigma Chemical Co., St Louis, MO, USA) and counted in a haemocytometer. The viability of sporozoites ranged from 90 to 100%.

### Animals

Male eight-week-old C57BL/6, 2DKO and 2DKO/KI mice (Taconic, Hudson, NY, USA) were used. On arrival, the animals were acclimated for seven days (quarantine). The animals were housed in a cage maintained in a room with a temperature range of 18-26°C [express in °C], 34-68% relative humidity and a 12-hr light/dark cycles. Food and water were provided *ad lib* during quarantine and throughout the study. The animals were fed a standard rodent maintenance diet. All animal studies were performed under IACUC approved protocols. All animal use, care and handling were performed in accordance with the current Guide for the Care and Use of Laboratory Animals (1996).

### Test agents and administration

The compounds tested in these experiments were dosed based on the body weight at the time of preparation of the suspension solution. The suspension solution of oral agents were prepared in 0.5% (w/v) hydroxyethyl cellulose and 0.2% (0.5% HECT, v/v) Tween-80 in distilled water, using homogenizer (PRO Scientific Inc, Monroe, CT, USA) with 10 mm open slotted generator to homogenize drug powder mixture at 20,000-22,000 rpm for 5 min in ice bath. A once-a-day, three-consecutive-day treatment regimen (−1, 0, 1 day) was used in all assessments. This volume was transferred to a 20-ml bottle, drawn into a 1-ml syringe, and delivered via intragastric feeder (18-gauge) to the designated recipient.

### IVIS spectrum

*In vivo* imaging of luciferase activity from luciferase expressing *P*. *berghei*-infected mice was performed using a Xenogen IVIS-200 Spectrum (Caliper Life Sciences, Hopkinton, MA, USA) *in vivo* imaging system. Mice were evaluated at 24, 48 and 72 hours post-sporozoite inoculation to determine liver- and blood-stage malaria infection. D-Luciferin potassium salt, (Xenogen, California and Goldbio, St Louis, MO, USA), the luciferase substrate, was intraperitoneally inoculated into mice at a concentration of 200 mg/kg 15 min before bioluminescence analysis. The mice were anaesthetized with isoflurane 3 min post-luciferin administration. The mice were then positioned ventral side up in the IVIS on the 37°C platform. The mice continued to receive isoflurane through the nose-cone delivery. The camera exposure time was 5 min for the 24, 48 and 72-hr time points with f-stop = 1 and large binning setting. Photons emitted from specific regions were quantified using Living Image® 3.0 software and total ROI was calculated. 3-D bioluminescent imaging tomography was performed with Living Image 3.0 software using sequential images taken with filters ranging from 580 to 660 nm.

## Results

### CYP2D6-mediated hydroxylation of primaquine

**Figure 1:**
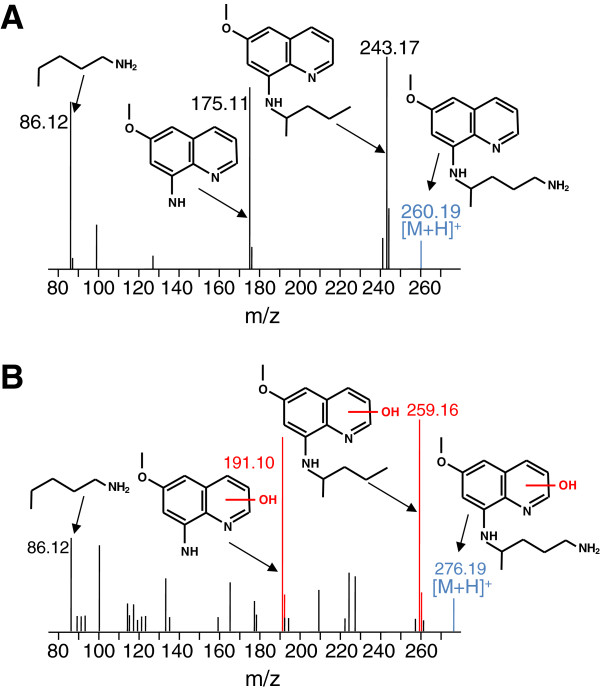
**CYP2D6**-**mediated hydroxylation of primaquine. A**. Shown is the MS^E^ fragmentation spectrum for primaquine. The (M + 1) charge state of primaquine is highlighted in blue. The major fragment ions of primaquine produced upon MS^E^ fragmentation are also shown along with their corresponding *m*/*z* values and structures. **B**. MS^E^ fragmentation of hydroxylated primaquine. Fragment ions utilized for localization of the hydroxylation to the quinoline core are indicated in red. It should be noted that MS^E^ fragmentation localizes this transformation to the quinoline core, and that the assignment shown is tentative.

In order to understand the role of CYP 2D6 in the biotransformation and bio-activation of primaquine, several experiments were conducted with primaquine and recombinant CYP2D6. Primaquine was incubated with CYP2D6 for 60 min with all necessary cofactors needed for CYP 2D6 activity. Incubations were then quenched with acetonitrile and the resulting metabolites formed were analysed by UPLC-MS analysis. The most abundant ions detected using the described experimental conditions were that of primaquine and a +16 Da modified metabolite. The corresponding MS^E^ spectra for primaquine and the metabolite are shown in Figures 1A and B respectively. Figure 1A shows the predominant fragment ions of primaquine and their corresponding *m*/*z* values. These fragments comprise the entire primaquine molecule including fragments of the quinoline core and the 8-amino side chain. The fragmentation pattern of the primaquine metabolite is shown in panel B. Fragments with +16 Da mass shifts were observed upon MS^E^ fragmentation and are highlighted in red. These fragments corresponded to the quinolone core of primaquine (191.10 and 259.16 *m*/*z*) and indicate that CYP 2D6 produces phenolic metabolites *in vitro*.

**Figure 2:**
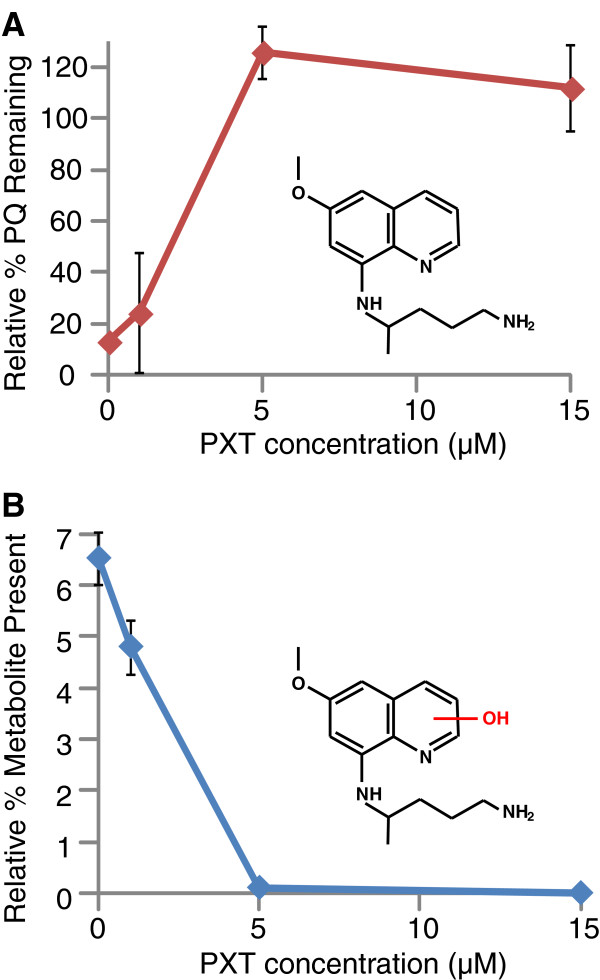
**Primaquine metabolism with paroxetine inhibition of CYP2D6. A**. Shown is the relative % primaquine remaining after 60-min incubations with CYP2D6 in the absence or presence of varying concentrations of paroxetine. **B**. Relative % hydroxylated metabolite present remaining after 60-min incubations with CYP2D6 in the absence or presence of varying concentrations of paroxetine. The structures of primaquine and the tentative hydroxylated metabolite are shown in each corresponding panel. Error bars were calculated from average values obtained from duplicate analyses.

To further probe the CYP 2D6-mediated metabolism of primaquine, the CYP 2D6 inhibitor paroxetine was utilized to determine if inhibition of the enzyme would prevent phenolic metabolite formation. Primaquine was incubated with CYP 2D6 in the absence or presence of varying concentrations of paroxetine. The relative % primaquine and the phenolic metabolite present were determined for each paroxetine incubation. The results for the paroxetine incubations are shown in Figure 2. Panel A shows the relative % primaquine remaining after 60 min with CYP 2D6. Under these conditions, primaquine was rapidly metabolized by CYP 2D6 in the absence of paroxetine, as less than 20% remained after 60 min. CYP 2D6-mediated metabolism of primaquine was significantly decreased upon incubation with increasing concentrations of paroxetine. In addition to monitoring primaquine parent loss, the formation of the phenolic metabolite described above was also monitored. Panel B shows the disappearance of this phenolic metabolite as a function of increasing paroxetine concentrations. These results indicate that CYP2D6 is important in the biotransformation of primaquine *in vitro* and that inhibiting the enzyme prevents formation of phenolic primaquine metabolites.

### Primaquine efficacy in CYP 2D knockout mice

**Figure 3:**
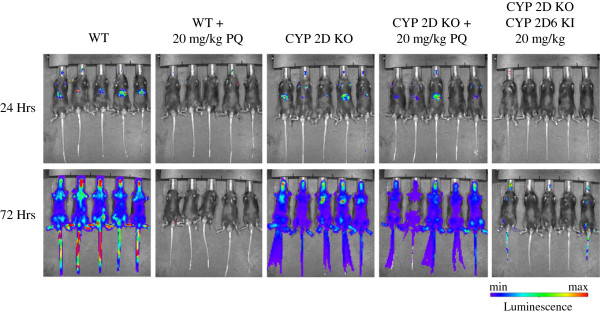
**Dissemination of malaria parasites by IVIS imaging of C57BL/6 Wild**-**type (WT)**, **CYP 2D knockout**, **and humanized CYP 2D knockout/CYP 2D6 knock**-**in mice at 24-hr and 72-hr post**-**inoculation with luciferase expressing *Plasmodium berghei*.** WT animals treated with 20 mg/kg x 3 days of oral PQ exhibited no parasitaemia at 24 or 72 hr. All five CYP 2D knockout mice exhibited liver stage parasitaemia at 24 hr which progressed to systemic infection by 72 hr. PQ efficacy was largely restored in mice in which the deleted CYP 2D cluster was replaced with human CYP 2D6.

**Figure 4:**
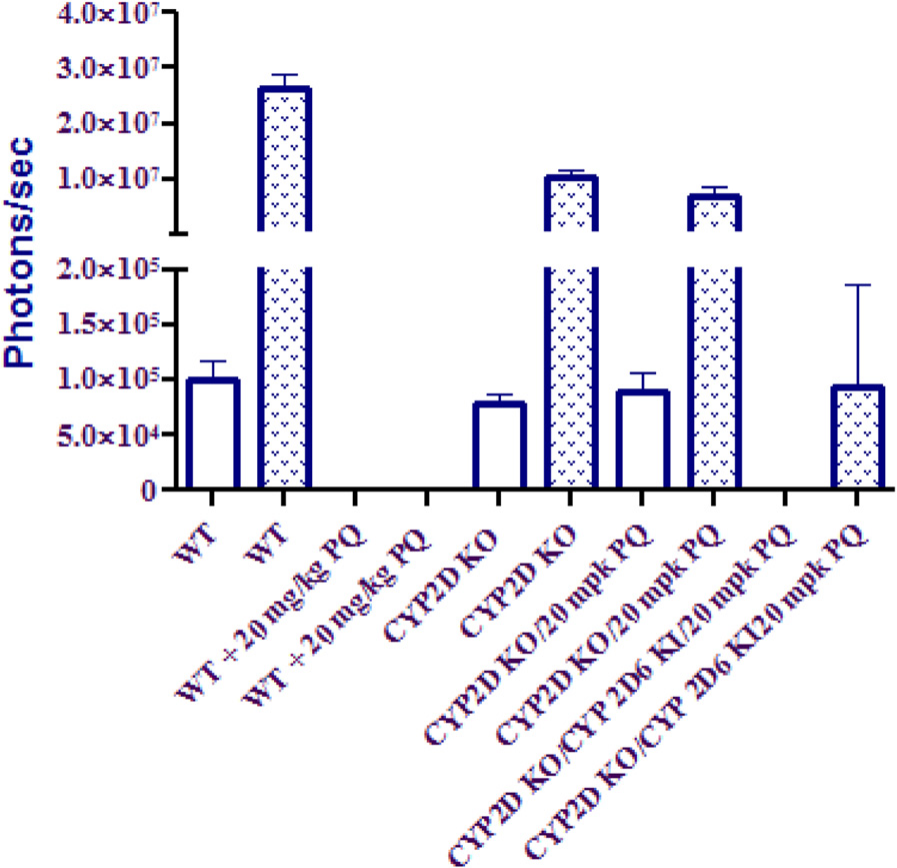
**Parasite dissemination by IVIS imaging of C57BL/6 WT, CYP 2D knockout, and humanized CYP 2D knockout/CYP 2D6 knock-in mice at 24-hr and 72-hr post**-**inoculation with luciferase expressing *Plasmodium berghei*.**

In order to assess the effects of CYP 2D metabolism on PQ efficacy, PQ was tested at its ED100 (20 mg/kg × 3 day PO) in C57BL/6 mice infected with luciferase expressing *P*. *berghei*. Of the five mice inoculated with sporozoites, none exhibited liver stage parasite signal out to 72 hr as compared with vehicle control (Figure 3). The same dose (20 mg/kg × 3 day PO) in mice containing a deletion of all nine mouse CYP 2D genes, including CYP 2D22 (the nearest murine ortholog to CYP 2D6) resulted in no cures. In addition to chemical inhibitors such as PXT to achieve diminished CYP 2D activity, knockout mice are presented here due to the poor specificity of chemical inhibitors *in vivo*. In order to determine whether this effect could be overcome via metabolic switching at higher doses, PQ was tested again at 40 mg/kg in the knockout mice, resulting in no cures (data not shown). Due to the uncertainty in the completeness of overlap between the activities of mouse CYP 2D22 and human 2D6, a humanized strain of mice in which the mouse 2D cluster has been removed and replaced with human 2D6 was inoculated with *P*. *berghei* sporozoites and treated with 20 mg/kg of PQ. In these animals, three of five mice had no detectable parasite burden in the liver at 72 hr as compared with vehicle control with a mean suppression of 99.3% (Figure 3). Ultimately, blood stage infections did appear in all five animals by day 8 post-inoculation, indicating incomplete inhibition of parasite growth. Figure 4 shows parasite burden as represented by luminescence from the luciferase expressing parasite for each dose group at 24 and 72 hr post-inoculation.

## Discussion

PQ therapy is a key component in the global fight against malaria, as it is the only approved drug indicated in the treatment of infections with species that show relapsing infections. As such it remains the standard of care for radical cure of *P*. *vivax* and *P*. *ovale*[12]. Given its gametocytocidal activity against stage 5 *Plasmodium falciparum* gametocytes, it is often considered in strategies for transmission blocking and malaria elimination [1,13,14]. Although not thoroughly understood, PQ efficacy is suspected to be linked to biotransformation via a CYP dependent pathway leading to the production of redox cycling metabolites [2,3,6,15]. Phenolic metabolites of PQ are the most likely candidates for such activity, and have frequently been linked to the associated haemolytic anaemia observed in G6PD-deficient individuals after PQ therapy [8,16-21]. Given the reactive nature of these metabolites and their demonstrated ability to redox cycle, they should be considered of potential importance to any mechanism of action dependent on the production of oxidative stress. It was recently demonstrated that these phenolic metabolites are predominantly generated via a CYP 2D6 dependent pathway [6].

Although CYP 2D6 is involved in the metabolism of 25% of all drugs on the market, it is known to be highly polymorphic, with more than 100 allelic variants reported [11,22,23]. Importantly, Bennett *et al*. reported two PQ failures in a *P*. *vivax* challenge which could be directly linked to two of these variants with diminished activity against hypnozoites, or the sleeping liver stages (submitted, *New England Journal of Medicine*). In the present study, efficacy of primaquine was assessed in a model of causal malaria prophylaxis (developing liver stages). The effect observed by Bennett *et al*. in this prophylaxis model using mice deficient in CYP 2D polymorphism was reproduced. In these animals, the 2D cluster is deleted, and can be replaced with a variant of human CYP 2D6 [11]. For the purposes of the present study, PQ efficacy against *P*. *berghei* was compared in the C57BL/6 background *versus* 2D knockout and humanized knockout knock-in. In this experiment, deletion of the CYP 2D cluster resulted in all five mice failing PQ therapy at the ED100 (20 mg/kg × 3 days PO) as determined in wild-type mice (Figure 3). Further, even twice the dose resulted in no cures in these animals (data not shown), suggesting that metabolites generated via 2D mediated pathways are unlikely to be produced by any other CYP through metabolic switching. This activity could, however, be partially restored by the introduction of human CYP 2D6 in the CYP 2D knockout/human CYP 2D6 knockin mice (Figure 3). In these animals, three of five showed no parasitaemia in 72 hr post-inoculation with an overall 99.3% mean suppression as compared to vehicle control. Ultimately all five animals presented with blood stage infections at day 8 post-infection. This could be due to the somewhat diminished activity of human CYP 2D6 in this model as compared to its mouse orthologue CYP 2D22, as Scheer *et al*. showed only about a 53% recovery of bufuralol 1’-hydroxylase activity in microsomes generated from livers of the humanized animals *versus* those from the wild-type [11]. This may well stem from intrinsic differences in activity toward common substrates between the two orthologues, and is not altogether surprising.

Here, presented are *in vitro* experiments that clearly demonstrate the effects of diminished CYP 2D6 activity on PQ metabolism. It was observed that the CYP 2D6 inhibitor paroxetine (PXT) inhibits parent loss of PQ when incubated with recombinant human CYP 2D6 in a dose-dependent manner. More importantly, it was also shown that the generation of phenolic metabolites (the major products of the metabolism of PQ by CYP 2D6), as illustrated in Figure 2, is also inhibited in a dose-dependent manner by PXT. It is important to note, that the P. berghei model utilized here contains no hypnozoites, and direct observations of PQ efficacy are only against primary developing liver stages. This activity is not necessarily related to PQ’s anti-hypnozoite activity, however PQ’s oxidative killing mechanism makes it likely that efficacy against all stages of the parasite are dependent on activation by metabolism. Taken into context with the murine data here reported, it is reasonable to conclude that: 1) PQ’s causal prophylactic anti-malarial efficacy is dependent on biotransformation by CYP 2D6; 2) phenolic metabolites generated by CYP 2D6 are responsible for this efficacy; and, 3) PQ causal prophylaxis will be impaired in patients with CYP 2D6 allelic variants of the poor and perhaps also the intermediate metabolizer type.

## Abbreviations

8AQ: 8-aminoquinoline; CYP: Cytochrome P450; PQ: Primaquine; CPQ: Carboxyprimaquine.

## Competing interests

The authors declare that they have no competing interests.

## Authors’ contributions

BSP participated in research design, performed data analysis, and contributed to the writing of the manuscript. SRM participated in research design, conducted experiments, performed data analysis, and contributed to the writing of the manuscript. XJ conducted experiments and contributed to the writing of the manuscript. GD participated in research design and contributed to the writing of the manuscript. JCS participated in research design, performed data analysis, and contributed to the writing of the manuscript. QL participated in research design and contributed to the writing of the manuscript. DC conducted experiments, performed data analysis, and contributed to the writing of the manuscript. QZ conducted experiments. GAR contributed to the writing of the manuscript. CO, CO, and LAW contributed new reagents and analytical tools and contributed to the writing of the manuscript. JB contributed to the writing of the manuscript. VM participated in research design, contributed new reagents and analytical tools, and contributed to the writing of the manuscript. All authors read and approved the final manuscript.
